# Seizure and Myelin Oligodendrocyte Glycoprotein Antibody-Associated Encephalomyelitis in a Retrospective Cohort of Chinese Patients

**DOI:** 10.3389/fneur.2019.00415

**Published:** 2019-04-26

**Authors:** Xiaonan Zhong, Yifan Zhou, Yanyu Chang, Jingqi Wang, Yaqing Shu, Xiaobo Sun, Lisheng Peng, Alexander Y. Lau, Allan G. Kermode, Wei Qiu

**Affiliations:** ^1^Department of Neurology, The Third Affiliated Hospital of Sun Yat-sen University, Guangzhou, China; ^2^Department of Medicine and Therapeutics, The Chinese University of Hong Kong, Shatin, China; ^3^Centre for Neuromuscular and Neurological Disorders, University of Western Australia, Perth, WA, Australia; ^4^Department of Neurology, Sir Charles Gairdner Hospital, Queen Elizabeth II Medical Centre, Perth, WA, Australia; ^5^Institute of Immunology and Infectious Diseases, Murdoch University, Perth, WA, Australia

**Keywords:** MOG-IgG, MOG antibody-associated encephalomyelitis, seizures, encephalopathy, demyelinating disease

## Abstract

**Background:** Myelin oligodendrocyte glycoprotein (MOG) antibody associated encephalomyelitis is increasingly being considered a distinct disease entity, with seizures and encephalopathy commonly reported. We investigated the clinical features of MOG-IgG positive patients presenting with seizures and/or encephalopathy in a single cohort.

**Methods:** Consecutive patients with suspected idiopathic inflammatory demyelinating diseases were recruited from a tertiary University hospital in Guangdong province, China. Subjects with MOG-IgG seropositivity were analyzed according to whether they presented with or without seizure and/or encephalopathy.

**Results:** Overall, 58 subjects seropositive for MOG-IgG were analyzed, including 23 (40%) subjects presenting with seizures and/or encephalopathy. Meningeal irritation (*P* = 0.030), fever (*P* = 0.001), headache (*P* = 0.001), nausea, and vomiting (*P* = 0.004) were more commonly found in subjects who had seizures and/or encephalopathy, either at presentation or during the disease course. Nonetheless, there was less optic nerve (4/23, 17.4%, *P* = 0.003) and spinal cord (6/16, 37.5%, *P* = 0.037) involvement as compared to subjects without seizures or encephalopathy. Most MOG encephalomyelitis subjects had cortical/subcortical lesions: 65.2% (15/23) in the seizures and/or encephalopathy group and 50.0% (13/26) in the without seizures or encephalopathy group. Cerebrospinal fluid (CSF) leukocytes were elevated in both groups. Subgroup analysis showed that 30% (7/23) MOG-IgG positive subjects with seizures and/or encephalopathy had been misdiagnosed for central nervous system infection on the basis of meningoencephalitis symptoms and elevated CSF leukocytes (*P* = 0.002).

**Conclusions:** Seizures and encephalopathy are not rare in MOG encephalomyelitis, and are commonly associated with cortical and subcortical brain lesions. MOG-encephalomyelitis often presents with clinical meningoencephalitis symptoms and abnormal CSF findings mimicking central nervous system infection in pediatric and young adult patients.

## Background

Immunoglobulin-G against myelin oligodendrocyte glycoprotein (MOG-IgG) is considered a potential demyelinating disease-associated autoantibody. Previous experimental studies have established MOG-IgG as a pathogenic antigen rather than an epiphenomenal bystander or a secondary immune reaction caused by previous demyelination (1–4). Although some cases of MOG-IgG positive patients fulfill the diagnostic criteria of neuromyelitis optica spectrum disorders (NMOSD), multiple sclerosis (MS), acute disseminated encephalomyelitis (ADEM), or other idiopathic inflammatory demyelinating diseases (IIDDs), there are no distinct types of IIDDs that can explain all presentations of MOG-IgG positive patients. Currently, most experts consider MOG-IgG-associated demyelination as an isolated disease entity distinct from both classic MS and aquaporin-4 (AQP4)-IgG-positive NMOSD (5–7).

MOG encephalomyelitis is associated with a wide spectrum of symptoms, including seizure and encephalopathy. Of note, seizure and encephalopathy have been recommended recently as typical clinical findings of MOG encephalomyelitis (8). In several case reports, MOG-IgG positive patients, who initially presented with optic neuritis (ON) (9) or ADEM (10), developed seizures in subsequent disease course. MOG-IgG positive patients often had an aggressive disease course with residual cognitive dysfunction (11). Several observational studies with small sample sizes reported the presence of seizures ranged from 14.70 to 36.36% in MOG-IgG positive patients (12, 13), and the main symptoms were generalized seizure with or without encephalopathy (12, 14). Nevertheless, studies with detailed description of the clinical, radiological, laboratory characteristics, and disease course of MOG-IgG positive patients with seizures and/or encephalopathy are lacking.

In our registry of patients with IIDDs, MOG-IgG positive patients with seizures and/or encephalopathy were also observed. In the present study, we investigated the clinical profiles of MOG-IgG positive patients with seizures and/or encephalopathy.

## Methods

### Subjects

Consecutive MOG encephalomyelitis patients who were seropositive for MOG-IgG and seronegative for AQP4-IgG were recruited from the Third Affiliated Hospital of Sun Yat-sen University in Guangzhou, China, between June 2015 and December 2017. These patients were prospectively enrolled and followed up for at least 1 year after diagnosis. Our hospital is a tertiary general hospital with a demyelinating disease center. Over 2,000 IIDDs patients, such as patients with MS, NMOSD, and ADEM and so on, are follow-up in outpatient per year, and about 300 newly diagnosed IIDDs patients attended every year. This study was approved by the Ethics Committees of the Third Affiliated Hospital of Sun Yat-sen University. Written informed consent was obtained from each participant.

We diagnosed MS, NMOSD, and ADEM according to the 2010 McDonald diagnostic criteria for MS (15), the 2015 Wingerchuk criteria for NMOSD (16), and the 2012 criteria for ADEM (17), respectively. Data of clinical presentation, initial clinical diagnosis, MOG-IgG serum titer, cerebrospinal fluid characteristics, MRI characteristics, treatments and prognosis were collected. A clinical relapse was defined as a sudden appearance of new symptoms lasting for at least 24 h, with an increase in the Expanded Disability Status Scale (EDSS) score over 1.0 and magnetic resonance imaging (MRI) showing the presence of enhanced lesions or new T2 lesions. The remission phase was defined as a period when the neurological condition of the patient had been stable for more than 3 months and the next relapse did not occur for at least a further 3 months. The EDSS score was evaluated at the nadir of disease recurrence when the patient first came to our hospital and at the last follow-up.

### Detection of MOG-IgG and AQP4-IgG

All subjects were tested for serum MOG-IgG and AQP4-IgG. Serum was collected at the nadir of clinical relapse when the subjects first came to one of our academic centers. MOG-IgG in serum was tested by an in-house, cell-based assay using live cells transfected with full-length human MOG, as we described in other published articles (18, 19). Full-length human MOG was subcloned into the pIRES2-EGFP plasmid. The purified plasmids were DNA sequenced and they were used to transiently transfect HEK293T cells using Lipofectamine2000 reagent, according to the manufacturer's instructions (Thermo Scientific, USA). Thirty-six hours after transfection, live cells were incubated at room temperature with centrifuged serum [1:50, diluted in Dulbecco's modified Eagle's medium (DMEM)] from patients and the control group for 30 min. After removing the media and washing with PBS, the HEK293T cells were fixed with 4% paraformaldehyde for 20 min and blocked with 5% goat serum for 30 min. Cells were then immunolabeled with an AlexaFluor 546 secondary antibody against human IgG (1:1,000; Thermo Scientific) for 1 h at room temperature. Images were acquired using a Zeiss Axiovert A1 fluorescence microscope (Supplement). Indirect immunofluorescence test systems for detecting human AQP4-IgG (Euroimmun Medizinische Labordiagnostika, Lübeck, Germany) were used according to the manufacturer's instructions.

### Study of Cerebrospinal Fluid (CSF) Samples

Lumbar puncture was performed in the acute phase of the disease. Cerebrospinal fluid (CSF) leukocyte count, total protein, and absence/presence of oligoclonal bands (OCB) were determined by the hospital laboratories.

### Magnetic Resonance Imaging Scanning

Brain and spinal cord MRI scans of subjects were performed for routine clinical diagnostic purposes using a 1.5 or 3.0 T Siemens system (Siemens). All MRI were performed using T1 with and without gadolinium enhancement and T2. In brain MRI scans T2 were also performed with T2 fluid-attenuated inversion recovery sequences. Lesions in the brain and spinal cord were scanned sagittally and axially, and the results were analyzed anonymously by two independent radiologists who were blinded to the subjects' clinical features. Lesions were scored as “large” if their size exceeded 2 cm in any plane.

### Statistical Analysis

For continuous variables, the data were reported as mean ± standard deviation or median with range. The Mann–Whitney *U*-test and chi-square test were used to compare clinical, laboratory and MRI data between subjects in different groups or subgroups. The Wilcoxon test was used to compare MOG-IgG titers at relapse and at remission within a group. Correlations between MOG-IgG titers and clinical data were analyzed using Spearman's correlation coefficient. All statistical analyses were performed using SPSS 23.0 software (SPSS Inc., Chicago, IL, USA) for Windows. Differences with *P* < 0.05 were considered statistically significant.

## Results

### Clinical Presentation

Overall, we recruited 58 subjects seropositive for MOG-IgG and seronegative for AQP4-IgG, including 23 (39.7%, 23/58) subjects with seizures and/or encephalopathy and 35 subjects without seizures or encephalopathy. The demographic and clinical features of the subjects were shown in Table 1.

**Table 1:** Comparison of clinical features between MOG-IgG positive subjects with or without seizures and/or encephalopathy.

	**Subjects with seizures or encephalopathy (*n* = 23)**	**Subjects without seizures or encephalopathy (*n* = 35)**
**Sex, male, N(%)**	12 (52%)	17 (49%)
**Onset age, years (range)**	12 (3–56)^*^	26 (3–63)
**Disease duration, months (range)**	34 (9–85)	22 (8–120)
**Follow-up, months (range)**	13 (5–85)	15 (4–50)
**Multiphasic disease course, N(%)**	18 (78%)^*^	16 (46%)
Number of attacks, N (range)	3 (2–8)	3 (2–5)
Time to second attack, months, median (range)	5 (1–48)	3 (1–48)
Annualized relapse rate, ARR median (range)	1.16 (0.67–2.77)	1.42 (0.38–3.75)
**With prodromal symptoms, N (%)**	7 (30%)	6 (17%)
**Manifestations at onset, N(%)**		
Seizures	11 (48%)^*^	0 (0%)
Disturbance of consciousness	6 (26%)^*^	0 (0%)
Psychiatric symptoms	2 (9%)	0 (0%)
Cognitive disorders	3 (13%)^*^	0 (0%)
Meningeal symptoms	3 (13%)^*^	0 (0%)
Optic nerve symptoms	4 (17%)^*^	21 (60%)
Spinal symptoms	5 (22%)	10 (29%)
Brainstem symptoms	2 (9%)	2 (6%)
Diencephalon	2 (9%)	0 (0%)
Cerebrum symptoms	5 (22%)	3 (9%)
Cerebellar symptoms	0 (0%)	0 (0%)
Fever	13 (57%)^*^	4 (11%)
Headache	13 (57%)^*^	4 (11%)
Dizziness	4 (17%)	3 (9%)
Nausea and Vomiting	5 (22%)^*^	0 (0%)
**Ever manifestations of full course, N(%)**		
Seizures	14 (61%)^*^	0 (0%)
Disturbance of consciousness	9 (39%)^*^	0 (0%)
psychiatric symptoms	4 (17%)^*^	0 (0%)
Cognitive disorders	4 (17%)^*^	0 (0%)
Meningeal symptoms	4 (17%)^*^	0 (0%)
Optic nerve symptoms	11 (48%)	24 (69%)
Spinal symptoms	7 (30%)	15 (43%)
Brainstem symptoms	4 (17%)	6 (17%)
Diencephalon	4 (17%)^*^	0 (0%)
Cerebrum symptoms	9 (39%)^*^	5 (14%)
Cerebellar symptoms	2 (9%)	0 (0%)
Fever	14 (61%)^*^	4 (11%)
Headache	14 (61%)^*^	4 (11%)
Dizziness	6 (26%)	3 (9%)
Nausea and Vomiting	11 (48%)^*^	1 (3%)
**EDSS score**		
EDSS score at peak stage (range)	5 (2–8) ^*^	3 (1–8.5)
EDSS score at last follow up (range)	0 (0–2) ^*^	1 (0–5)
**Autoantibody**, ***N*** **(%)**		
Concomitant autoantibody	3 (13%)	5 (14%)
Coexisting autoimmune disease^#^	1 (4%)	1 (3%)
**MOG-Ab titer**, ***N*** **(%)**		
1:25-1:100	10 (43%)	16 (46%)
1:320-1:640	9 (39%)	14 (40%)
≧ 1:1280	4 (17%)	5 (14%)
**AQP4-IgG**, ***N*** **(%)**	0(0%)	0(0%)

*Time to second attack and annualized relapse rate refers to multiphasic patients*.

*EDSS, expanded disability status scale*.

*NA, not available*.

# *Coexisting autoimmune disease refers to autoimmune disease other than IIDDs*.

* *P < 0.05*.

Subjects with seizures and/or encephalopathy had a younger onset age and a higher EDSS score at the nadir stage of disease compared to those without seizure and/or encephalopathy. At disease onset, the percentages of meningeal irritation (including nuchal rigidity, Brudzinski sign, or Kerning sign), fever, headache, nausea and vomiting were significantly higher in subjects who experienced seizures and/or encephalopathy. But there was less optic nerve involvement in the patients had seizures and/or encephalopathy.

### Seizures and Encephalopathy in MOG-IgG Positive Subjects

During the course of the disease, seizure was observed in 14 subjects among the whole 58 patients recruited in our study (all in the MOG encephalomyelitis with seizures and/or encephalopathy group): 11 (79%) subjects had seizure as the first symptom, and 3 (21%) developed seizure during the subsequent relapse at 5, 29, and 42 months, respectively after the disease onset. The types of seizure were generalized tonic-clonic seizure (*n* = 7, 50%), focal seizure with secondary generalization (*n* = 5, 36%), complex partial seizure with alternated conscious and facial twitching (*n* = 1, 7%), and simple partial seizure with focal left arm twitching (*n* = 1, 7%).

Encephalopathy was observed in 13 subjects during the period of the disease. This symptom was the first symptom in 10 (77%) subjects; subjects had disturbance of consciousness with varying degrees of somnolence and stupor, psychiatric symptoms including hallucinations, confused speech and apathy, and, cognitive disorders including memory impairment and acalculia.

Electroencephalogram (EEG) was abnormal among 15 (25.9%) subjects, including slowed background (theta to delta rhythm), intermittent low amplitude fast waves, focal sharp-wave complex and asymmetry focal slow waves.

### Initial Clinical Diagnosis

**Figure 1:**
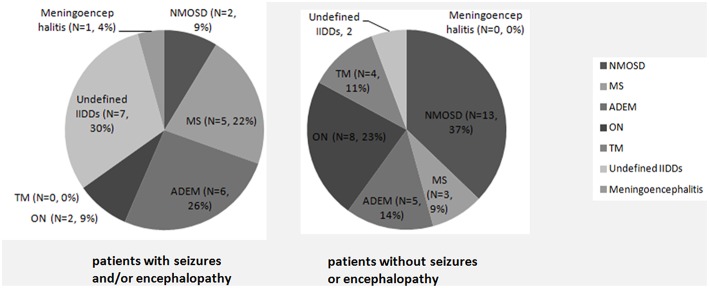
Initial clinical diagnosis in MOG-IgG positive patients with or without seizures and/or encephalopathy. A significantly higher proportion of subjects with seizures and/or encephalopathy were diagnosed as non-specific IIDDs (*P* = 0.030), while NMOSD were commonly diagnosed among subjects without seizures or encephalopathy (*P* = 0.035).

A significantly higher proportion of subjects suffered from seizures and/or encephalopathy were diagnosed as non-specific IIDDs, while NMOSD was a common diagnosis in the patients who did not subject from seizures or encephalopathy (Figure 1).

### MOG-IgG Serum Titer

**Figure 2:**
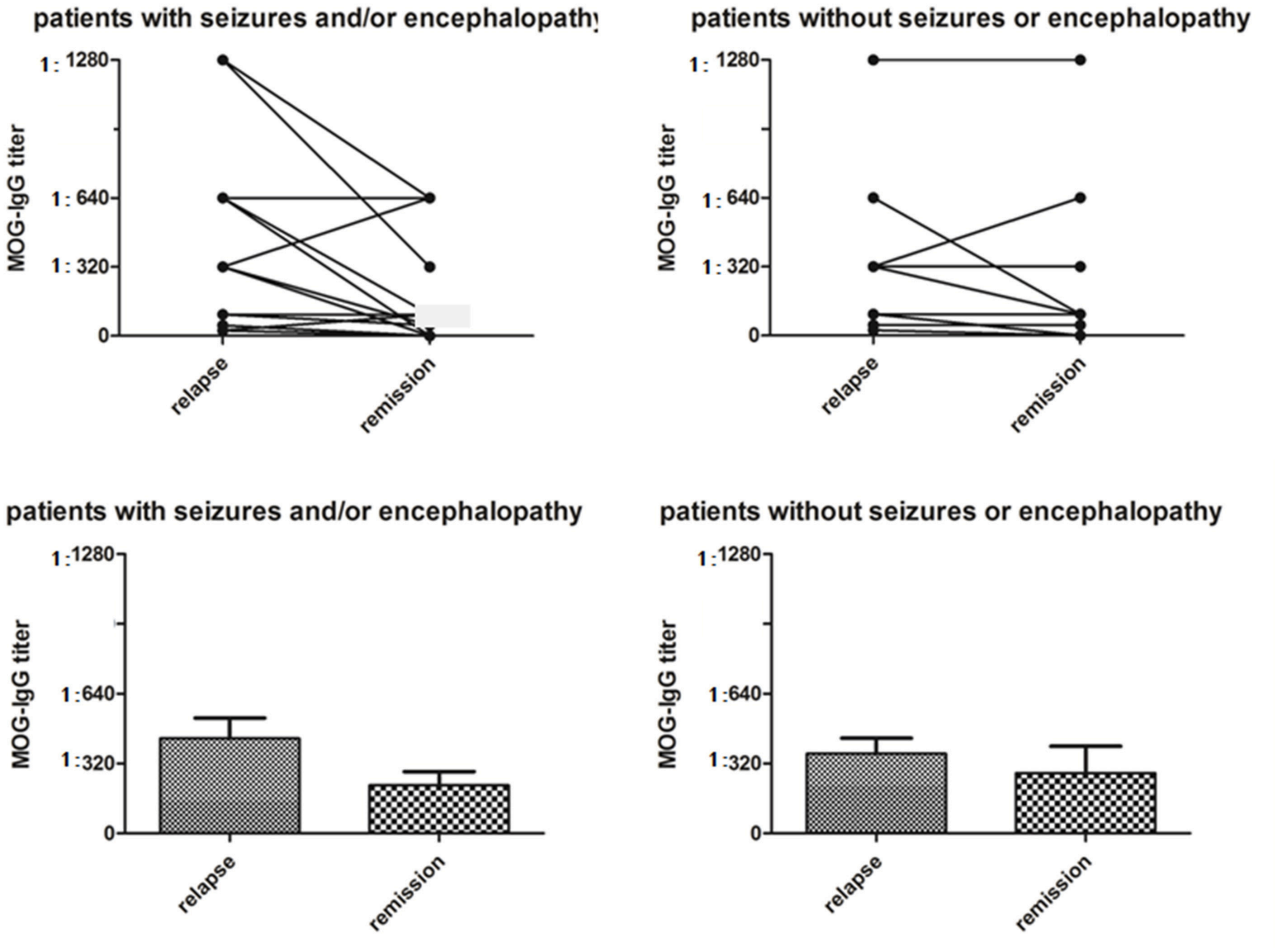
Serum MOG-IgG titers in MOG-IgG positive patients with or without seizures and/or encephalopathy. In the seizure and/or encephalopathy group, MOG-IgG titers were significantly higher at the nadir stage of relapse when compared with titers at last follow-up (*P* = 0.021), although there was no difference in serum MOG-IgG titers between relapses and remission in the without seizures or encephalopathy group (*P* = 0.080).

The median serum MOG-IgG titer at the nadir stage of disease was 1:320 (range 1:25–1:1280). There was no difference in the MOG-IgG titer between subjects with and without seizure and/or encephalopathy (Table 1, Figure 2).

In the seizures and/or encephalopathy group, the MOG-IgG titer at the peak stage was positive related with EDSS score at last follow-up (Table 2).

**Table 2:** Correlations between MOG-IgG titers at relapse and clinical features in MOG-IgG positive patients.

	**Patients with seizures and encephalopathy (*n* = 23)**	**Patients without seizures or encephalopathy (*n* = 35)**
	***r* value**	***r* value**
MOG-IgG titers at last follow up	0.558^*^	0.921^*^
EDSS score at peak stage	0.150	0.415^*^
EDSS score at last follow up	0.494^*^	−0.188
Annual relapse rate	−0.020	−0.024
CSF leukocyte	−0.134	0.368
CSF total protein	−0.188	0.462^*^

*r: correlation coefficient*.

*CSF: cerebrospinal fluid; EDSS, expanded disability status scale; MOG-IgG, immunoglobulin G against myelin oligodendrocyte glycoprotein*.

*NA, not available*.

* *P < 0.05*.

### Other Autoantibodies and Autoimmune Disease

Rheumatoid and thyroid autoantibodies were found in a small number of subjects, including antinuclear antibody (3/58, 5%), anti-Sjogren syndrome A antibody (SSA) (2/58, 3%), anti-thyroid peroxidase antibodies (aTPO) and/or antithyroglobulin antibodies (aTG) (5/58, 9%). Concurrent systemic autoimmune diseases were found in 2 subjects; one had systemic vasculitis and the other has autoimmune hyperthyroidism (Table 1).

### CSF Investigation

Records of CSF were available for analysis in 45 (78%, 45/58) subjects, including 18 (78%, 18/23) subjects in the seizures and/or encephalopathy group and 27 (77%, 27/35) subjects in the without seizures or encephalopathy group. Some CSF data in remaining 13 (22%, 13/58) subjects were examined in the local hospitals which the patients had visited before they came to our hospital and were not offered to us. CSF leukocytosis was noted in 61% (11/18) subjects subjected from seizures and/or encephalopathy and 41% (11/27) subjects who did not have seizures or encephalopathy, and there was no statistical significance in the levels of CSF leukocytosis between these groups. No difference was found in the CSF protein concentration between the two groups. Oligoclonal band was present in 7 (12%, 7/58) subjects, and no difference was noted among those with or without seizure and/or encephalopathy (Table 3).

**Table 3:** Comparison of CSF features between MOG-IgG positive patients with or without seizures and/or encephalopathy.

	**Patients with seizures or encephalopathy (*n* = 18)**	**Patients without seizures or encephalopathy (*n* = 27)**
Presence of oligoclonal bands, N (%)	3 (16.7%)	4 (14.8%)
Leukocyte count, x10^6^/L, median (range)	21 (0–457)	2 (0–314)
Total protein (mg/L), median (range)	0.25 (0.09–1.06)	0.28 (0.07–0.99)

### MRI Findings

**Figure 3:**
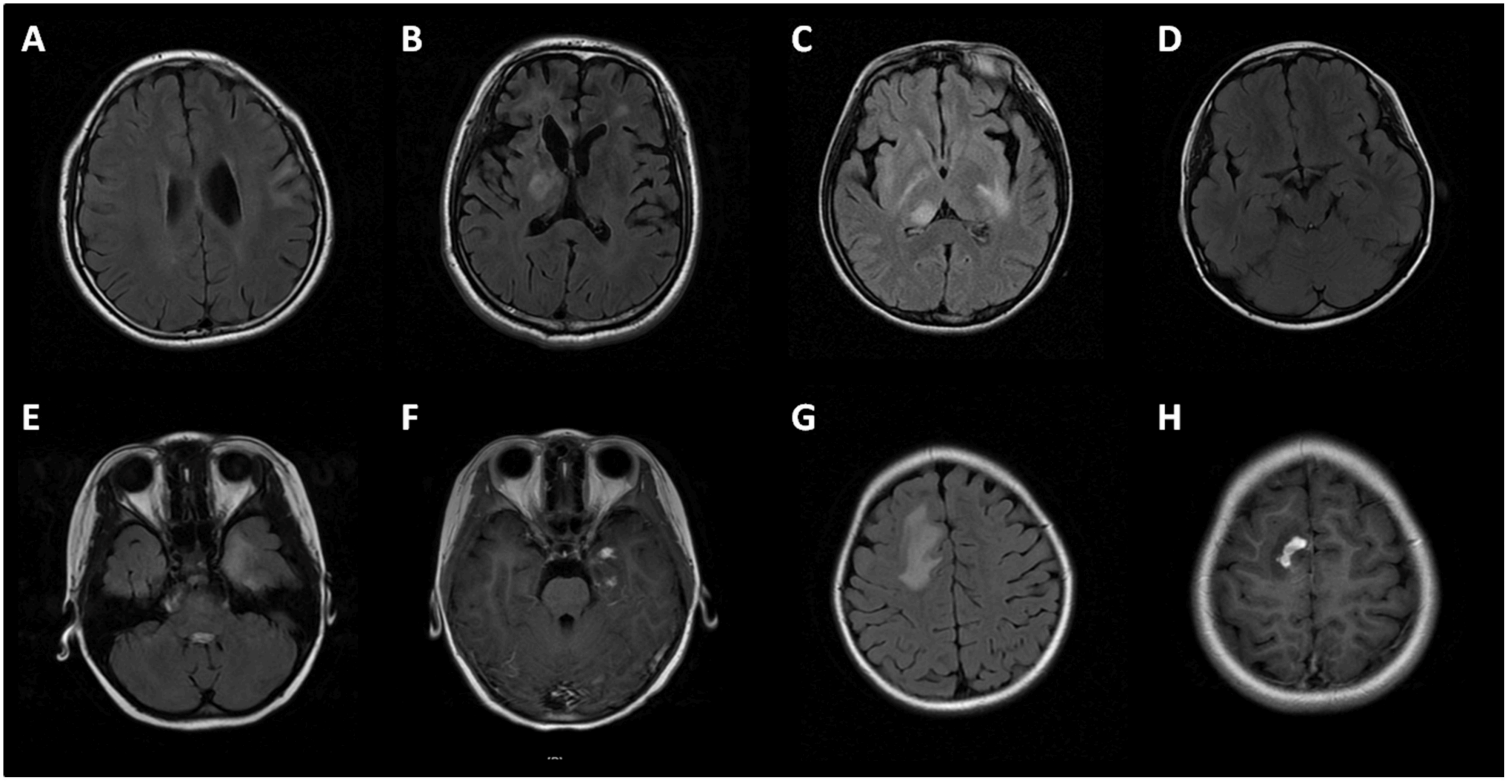
Brain MRI of MOG-IgG positive patients with seizures and/or encephalopathy. **(A)** Cortical and subcortical lesions; **(B)** Deep white matter lesions and periventricular lesions; **(C)** Thalamus lesions; **(D)** Cerebral peduncle lesions; **(E,G)** Large white matter lesions of a patient in temporal lobe and parietal lobe, with prominent gadolinium enhancement **(F,H)**.

Brain MRI was performed in 46 (79%, 46/58) subjects. In MOG-IgG positive subjects with seizures and/or encephalopathy, the lesions in cortical/subcortical (15/23, 65%), white matter (including periventricular and corpus callosum, 21/23, 91%), deep gray matter (including thalamus and basal ganglia, 13/23, 57%), and infratentorial (including cerebral peduncle, brain stem and cerebellum, 14/23, 60.9%) areas were involved (Figure 3). There was a higher proportion of deep white matter and cerebral peduncle in the seizures and/or encephalopathy group.

Spinal MRI was performed in 40 (69%, 40/58) subjects, including 16 subjects with seizures and/or encephalopathy and 24 subjects without seizures or encephalopathy. Less involvement of the spinal cord was found in the seizures and/or encephalopathy group (Table 4).

**Table 4:** Comparison of MRI features between MOG-IgG positive patients with or without seizures and/or encephalopathy.

	**Patients with seizures and encephalopathy**	**Patients without seizures or encephalopathy**
**BRAIN MRI**, ***N*** **(%)**		
Total abnormal	21 (91%)	22 (85%)
Frontal lobe	18 (86%)	12 (55%)
Parietal lobe	13 (62%)	12 (55%)
Temporal lobe	11 (52%)	7 (32%)
Hippocampus	3 (14%)	1 (5%)
Occipital lobe	7 (33%)	5 (23%)
Insula	7 (33%)	2 (9%)
Meninges	3 (14%)	2 (9%)
Cortical	7 (33%)	4 (18%)
Subcortical	14 (67%)	12 (55%)
Deep white matter	20 (95%)^*^	10 (45%)
Periventricular	14 (67%)	9 (41%)
Corpus callosum	7 (33%)	4 (18%)
Thalamus	7 (33%)	5 (23%)
Hypothalamus	0 (0%)	1 (5%)
Basal ganglia	8 (38%)	6 (27%)
Internal capsule	1 (5%)	2 (9%)
cerebral peduncle	7 (33%)^*^	0 (0%)
Midbrain	4 (19%)	3 (14%)
Pons	6 (29%)	4 (18%)
Medulla	4 (19%)	2 (9%)
Cerebellum	7 (33%)	2 (9%)
Extensive white matter lesions	8 (38%)	3 (14%)
**SPINAL MRI**, ***N*** **(%)**		
Total abnormal	6 (37.5%)^*^	17 (71%)
LETM	4 (67%)	8 (47%)
Distribution		
Cervical	5 (83%)	16 (94%)
Thoracic	4 (67%)	11 (65%)
Lumbar	1 (17%)	2 (12%)
**OPTIC NERVE MRI**, ***N*** **(%)**		
Total abnormal	6 (75%)	9 (69%)
Bilateral ON	2 (33%)	7 (78%)
LEON	2 (33%)	4 (44%)

*LEON, Longitudinally extensive optic neuritis; LETM, Longitudinally extensive transverse myelitis*.

*NA, not available*.

* *P < 0.05*.

### Clinical Course Over Time

After followed up for at least 1 year, subjects suffered from seizures and/or encephalopathy had more multiphasic disease course compared to those without seizure and/or encephalopathy. At the last follow-up, complete recovery (EDSS 0) was noted in over half (*n* = 14, 61%) of the subjects who had seizures and/or encephalopathy, and a smaller proportion of benign prognosis was observed in the counterparts without seizures and/or encephalopathy (*n* = 13, 37%). Throughout the period of the disease, the percentages of meningeal irritation, fever, headache, nausea and vomiting, diencephalon, and cerebrum symptoms were significantly higher in subjects with seizures and/or encephalopathy than those without. The proportions of number of attacks, time to the second attack and annual relapse rate were not significantly different between the two groups.

We evaluated the longitudinal changes in serum MOG-IgG titres during the remission phase at the last follow-up in 17 (73.9%) subjects who had seizure and/or encephalopathy and 14 (40.0%) subjects not experiencing these symptoms; MOG-IgG titer decreased 65% and 57% in the two groups, respectively. In the seizures and/or encephalopathy group, MOG-IgG titers were significantly higher at the nadir stage compared with last follow-up (Figure 2).

### Treatments

All subjects received treatment for acute attacks. High-dose intravenous methylprednisolone (10–20 mg/kg/d for 3–5 d) was used in 22 (96%) subjects with seizures and/or encephalopathy, and 5 (22%) received intravenous immunoglobulin. For subjects without seizure and/or encephalopathy, intravenous methylprednisolone was used in 35 (100%) subjects, and 3 (9%) received intravenous immunoglobulin. Low-dose oral glucocorticoid (4–8 mg qd/qod) for maintenance was used in 10 (44%) subjects suffered from seizures and/or encephalopathy and 20 (57%) subjects who did not have seizure and/or encephalopathy. Long-term immunosuppressive or immunomodulatory treatments were used in 7 (30%) of subjects with seizures and/or encephalopathy: rituximab (*n* = 3, 13%), azathioprine (*n* = 2, 9%), mycophenolate mofetil (*n* = 1, 4%), and tacrolimus (*n* = 1, 4%). For subjects without seizure and/or encephalopathy, 12 (34%) subjects were treated with immunosuppressive or immunomodulatory treatments: azathioprine (*n* = 7, 20%), mycophenolate mofetil (*n* = 2, 6%), rituximab (*n* = 1, 3%), tacrolimus (*n* = 1, 3%), and cyclophosphamide (*n* = 1, 3%).

The two subgroups have similar response to the corticosteroids and immunosuppression. In the seizure and/or encephalopathy group, 18 subjects had multiphasic disease course. And 10 of these 18 subjects were treated by low-dose oral glucocorticoid at the early stage of disease, but 40% (4/10) of them were suffered from relapse and started the combination therapy of oral glucocorticoid and immunosuppressive; however, 1 of the patients receive combination therapy still experienced treatment failure. Another 3 subjects with a high relapse rate did not recurrence after immunosuppressive treatment. Among the 16 subjects without seizure and/or encephalopathy, low-dose corticoid was utilized in 9 subjects, and 33.3% (3/9) of them relapse but refuse to immunosuppression, with 2 of these 3 subjects experienced recurrence after steroid withdrawal. No relapse was observed after immunosuppression was used in 7 subjects.

In the seizures and/or encephalopathy group, antiviral drugs were used in 4 (17%) subjects who were initially suspected to have CNS viral infection, antibiotics were used in 5 (22%) subjects initially suspected to have a CNS bacterial infection, and 1 (4%) subject initially diagnosed as tuberculosis meningoencephalitis was treated with anti-tuberculosis drugs.

Some subjects (10/14, 71%) with seizure were treated with anti-epileptic drugs, including levetiracetam (*n* = 4, 29%), oxcarbazepine (*n* = 3, 21%), carbamazepine (*n* = 2, 14%), sodium valproate (*n* = 2, 14%), and nitrazepam (*n* = 1, 7%).

### Subgroup Analysis of Subjects With Seizures

Among the 14 (24%) subjects who developed seizure, 11 (79%) subjects had seizure as their first symptoms. These subjects were younger (*P* < 0.001) and were associated with more clinical relapses (*P* = 0.007). In addition to seizure, these subjects commonly presented with disturbance of consciousness, meningism, fever, headache, nausea and vomiting, as well as cognitive and brainstem symptoms (*P* < 0.05) (Table 1). CSF leukocytosis and cortical/subcortical brain lesions on MRI were noted in this subgroup (*P* < 0.05) (Tables 3, 4).

### Subgroup Analysis of Subjects With Meningoencephalitis

A small subgroup of subjects (*n* = 7, 12%) presented with symptoms suggesting CNS infection, including fever, headache, nausea, meningism, seizures, and encephalopathy. Antiviral or antibacterial treatments were prescribed. The CSF showed raised opening pressure (median 205 mm) and marked leukocytosis (median 177 × 10^6^/L), and raised total protein (median 0.49 mg/L).

## Discussion

We have presented the clinical features of a cohort of Chinese patients with MOG-associated encephalomyelitis from Guangdong, China. We found that seizures and/or encephalopathy were commonly seen in pediatric and young adult patients with MOG-IgG, often complicating with a relapsing disease course. Unlike older patients with classical optic neuritis, myelitis, or brainstem syndromes, these patients were often diagnosed as CNS infection due to clinical, radiological, and CSF findings. MOG encephalomyelitis should be considered to be a differential diagnosis in these patients.

Consistent with previous studies, ON, myelitis, and/or brainstem symptoms were the predominant clinical features in most adult MOG-encephalomyelitis patients, whilst in children and younger adults there was a shift toward ADEM imitation (8, 18, 20–22), with encephalopathy as a clinical characteristic.

In our study, the percentage of subjects with seizures and/or encephalopathy was 40%, which is higher than most of the previous studies, and is more consistent with a recent study by Gutman et al. (13). However, our study had a much larger sample size (*n* = 58). Therefore, we speculate that the proportion of seizures and encephalopathy in MOG encephalomyelitis patients is higher than originally thought, but in the past, these patients might be diagnosed as CNS infection rather than immune mediated encephalomyelitis. Moreover, most of these patients had seizures (79%) and/or encephalopathy (77%) as their first symptoms. Furthermore, generalized seizure was the main type of seizure in our study, which is consistent with a study by Ogawa et al. (14). In our cohort, 42% subjects had focal seizure with secondary generalization, suggesting that epileptogenic regions, such as the cortex and temporal lobe, may be affected. This was supported by the finding of cortical lesions on MRI brain in 40% of our cohort.

In addition, we added further knowledge to previous studies by showing that meningoencephalitis symptoms, including fever, headache, nausea/vomiting, meningeal irritation, and CSF leukocytosis were common in MOG encephalomyelitis patients who were suffered from seizures and/or encephalopathy. Some of these symptoms had been noticed in various previous studies; however, most of the studies did not discuss the symptoms with seizures and/or encephalopathy meanwhile (23). Often, these patients may have delayed immunosuppressive treatment because of being misdiagnosed as CNS infection. Therefore, young patients with fever and meningoencephalitis should also be evaluated for possible MOG encephalomyelitis. Immunosuppressive treatment could be commenced if CSF microbiological results were negative and MOG-IgG was positive, which may improve clinical prognosis.

Another finding was the multiphasic course of patients who were subjected to seizures and/or encephalopathy. Similarly, disease relapse was reported in all 5 MOG-IgG positive patients with seizures in a study by Hamid et al. (12). Yet our patients who had not experienced seizures or encephalopathy had a lower multiphase ratio which were analogous to the previous researches (23–25). Thus, maintenance immunomodulation treatment should be used to prevent relapse in MOG encephalomyelitis patients who had seizures and/or encephalopathy.

From another perspective, part of the clinical features of our MOG encephalomyelitis patients with seizures and/or encephalopathy was close to that of MOG encephalomyelitis patients without seizures or encephalopathy, suggesting that these two subgroups were the same disease. For instance, our MOG encephalomyelitis patients who were suffered from seizures and/or encephalopathy also had a less female dominance, a relative lower coexisting autoimmunity rate, a better response to steroid and immunosuppression and a more benign prognosis compared with NMOSD and other IIDDs (21, 23–26). Moreover, these patients had a correlation between disease condition and MOG-IgG antibody titer which was the same as the counterparts who did not experience seizures or encephalopathy (27).

Seizures and encephalopathy, which suggest neuronal damage on top of demyelinating disease affecting the white matter, further support MOG encephalomyelitis as a broader disease entity. The observations in the present study suggest that MOG encephalomyelitis with seizures and/or encephalopathy may be a distinct clinical disease entity in addition to commonly recognized demyelinating diseases.

## Conclusions

Seizures and encephalopathy are common among subjects with MOG-associated encephalomyelitis, and may be associated with cortical and subcortical brain lesions. Young subjects with high titers of MOG-IgG may present with meningoencephalitis mimicking CNS infection.

## Ethics Statement

This study was carried out in accordance with the recommendations of Ethics Committees of the Third Affiliated Hospital of Sun Yat-sen University with written informed consent from all subjects. All subjects gave written informed consent in accordance with the Declaration of Helsinki. The protocol was approved by the Ethics Committees of the Third Affiliated Hospital of Sun Yat-sen University.

## Author Contributions

XZ, WQ, LP, AL, and AK determined the study design and performed the study. XZ, YZ, and YC were responsible for data collection. XZ, JW, YS, and XS analyzed the data. XZ and WQ drafted the manuscript. All authors read, critically revised, and approved the final manuscript.

### Conflict of Interest Statement

The authors declare that the research was conducted in the absence of any commercial or financial relationships that could be construed as a potential conflict of interest.

## Abbreviations

ADEM, acute disseminated encephalomyelitis AQP4: aquaporin-4
ATM: acute transverse myelitis
CNS: central nervous system
CSF: cerebrospinal fluid
EDSS: expanded disability status scale
IIDDs: idiopathic inflammatory demyelinating diseases
MOG: myelin oligodendrocyte glycoprotein
MOG-IgG: immunoglobulin G against myelin oligodendrocyte glycoprotein
MRI: magnetic resonance imaging
MS: multiple sclerosis
NMOSD: neuromyelitis optica spectrum disorders
OCB: oligoclonal bands
ON: optic neuritis
LEON: Longitudinally extensive optic neuritis
LETM: Longitudinally extensive transverse myelitis.
