# Supplementary material for: Seizure and Myelin Oligodendrocyte Glycoprotein Antibody-Associated Encephalomyelitis in a Retrospective Cohort of Chinese Patients
Source: Front Neurol. 2019 Apr 26;10:415. doi: 10.3389/fneur.2019.00415 (PMC6497765; doi:10.3389/fneur.2019.00415)
Supplement: Supplementary file 1 [file Data_Sheet_1.docx]

**Seizure and Myelin Oligodendrocyte Glycoprotein Antibody-associated Encephalomyelitis in a retrospective cohort of Chinese Patients**

**Supplement**

**
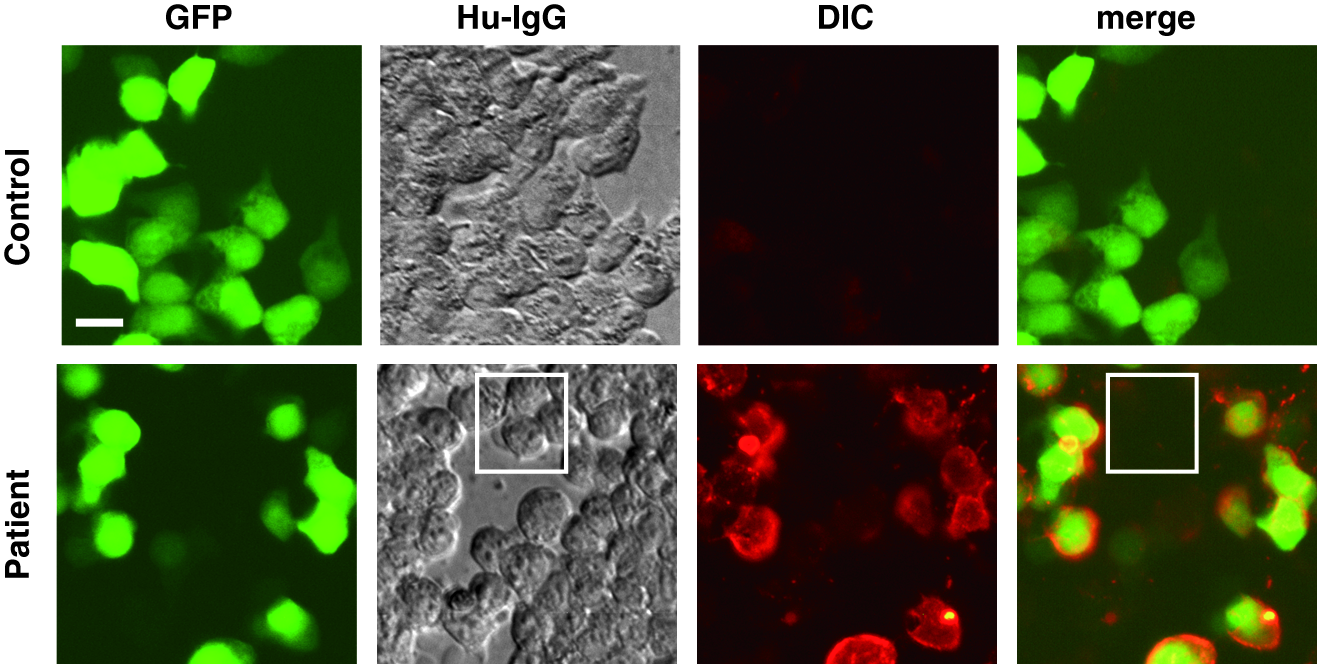
**

**Supplement. Serum antibodies to myelin oligodendrocyte glycoprotein (MOG) as detected by Cell-based assays**

MOG-IgG in serum was tested by an in-house, cell-based assay using live cells transfected with full-length human MOG, as we described in other published articles. Full-length human MOG was subcloned into the pIRES2-EGFP plasmid. The purified plasmids were DNA sequenced and they were used to transiently transfect HEK293T cells using Lipofectamine2000 reagent, according to the manufacturer's instructions (Thermo Scientific, USA). Thirty-six hours after transfection, live cells were incubated at room temperature with centrifuged serum (1:50, diluted in Dulbecco’s modified Eagle’s medium (DMEM)) from patients and the control group for 30 minutes. After removing the media and washing with PBS, the HEK293T cells were fixed with 4% paraformaldehyde for 20 minutes and blocked with 5% goat serum for 30 minutes. Cells were then immunolabeled with an AlexaFluor 546 secondary antibody against human IgG (1:1000; Thermo Scientific) for 1 h at room temperature. Images were acquired using a Zeiss Axiovert A1 fluorescence microscope. The patient’s IgG bound to MOG-transfected cells but not to un-transfected cells (indicated with square box). Serum IgG from control group does not bind. Scale bar:20 µm
